# The Synergistic *In Vitro and In Vivo* Antitumor Effect of Combination Therapy with Salinomycin and 5-Fluorouracil against Hepatocellular Carcinoma

**DOI:** 10.1371/journal.pone.0097414

**Published:** 2014-05-09

**Authors:** Fan Wang, Weiqi Dai, Yugang Wang, Miao Shen, Kan Chen, Ping Cheng, Yan Zhang, Chengfen Wang, Jingjing Li, Yuanyuan Zheng, Jie Lu, Jing Yang, Rong Zhu, Huawei Zhang, Yingqun Zhou, Ling Xu, Chuanyong Guo

**Affiliations:** 1 Department of Gastroenterology, Shanghai Tenth People’s Hospital, Tongji University of Medicine, Shanghai, PR China; 2 Department of Gastroenterology, Shanghai Tongren Hospital, Jiaotong University of Medicine, Shanghai, PR China; 3 Department of Gastroenterology, Clinical Medicine of Shanghai Tenth People’s Hospital, Nanjing Medical University, Shanghai, PR China; 4 Department of Gastroenterology, The First Hospital Affiliated to Suzhou University, Suzhou, PR China; Taipei Medicine University, Taiwan

## Abstract

Hepatocellular carcinoma (HCC) is one of the few cancers in which a continuous increase in incidence has been observed over several years. Drug resistance is a major problem in the treatment of HCC. In the present study, we used salinomycin (Sal) and 5-fluorouracil (5-FU) combination therapy on HCC cell lines Huh7, LM3 and SMMC-7721 and nude mice subcutaneously tumor model to study whether Sal could increase the sensitivity of hepatoma cells to the traditional chemotherapeutic agent such as 5-FU. The combination of Sal and 5-FU resulted in a synergistic antitumor effect against liver tumors both *in vitro and in vivo*. Sal reversed the 5-FU-induced increase in CD133(+) EPCAM(+) cells, epithelial–mesenchymal transition and activation of the Wnt/β-catenin signaling pathway. The combination of Sal and 5-FU may provide us with a new approach to reverse drug resistant for the treatment of patients with HCC.

## Introduction

Hepatocellular carcinoma (HCC) is one of the few cancers in which a continuous increase in incidence has been observed over several years [1]. According to the Barcelona Clinic Liver Cancer (BCLC) diagnostic and treatment strategy, chemotherapy is the best option for advanced stage tumors or HCC with extrahepatic diseases [2]. However, multidrug resistance has been identified as a major factor in the poor prognosis of patients suffering from advanced staged HCC. In recent years, investigations have focused on the factors contributing to drug resistance in HCC and possible approaches towards overcoming this therapeutic challenge have aroused interest in many researchers.

To date, more than 100 drugs are used to treat HCC. The chemotherapeutic drug 5-fluorouracil (5-FU), is effective against various types of cancer, including colorectal [3], breast [4], stomach and gullet cancer [5], it is also the optimal drug for the treatment of HCC [6]. However, the rapid development of acquired resistance to 5-FU has limited its clinical usage [7]–[9]. As the traditional chemotherapeutic agent available for the treatment of advanced HCC, 5-FU monotherapy is not very effective and is associated with multiple adverse events and drug resistance.

The recent emergence of the cancer stem cells (CSCs) concept suggests why treatment with chemotherapy such as 5-FU may often seem to be initially successful, but eventually results in not only a failure to eradicate the tumor, but also possible tumor relapse [10], [11]. Commonly used anticancer drugs such as 5-FU are effective against HCC by targeting the rapidly proliferating and differentiating HCC cells which constitute the bulk of the tumor. These therapies may spare CSCs, allowing for regeneration of the tumor [12], [13]. Recently, a robotic high-throughput screening approach was used to evaluate approximately 16,000 compounds from chemical libraries for activity against human breast CSCs, and only salinomycin (Sal) which was originally used to kill bacteria, fungi, and parasites, markedly and selectively reduced the viability of breast CSCs [14]. Then, Tang et al. [15] demonstrated that Sal is an effective inhibitor of osteosarcoma stem cells, Kusunoki et al. [16] reported that Sal had an inhibitory effect on the properties of endometrial CSCs. In our previous studies [17], [18] we showed that Sal could down-regulate the proportion of CD133+ cell subpopulations which have stem cell properties in HCC and pancreatic cancer.

Based on the idea that cancer cells at different degrees of differentiation are targeted by 5-FU and Sal, we combined Sal and 5-FU to determine whether this combination could increase HCC sensitivity to 5-FU and eradicate HCC cells. The possible mechanisms of this effect were also investigated.

## Materials and Methods

### 2.1 Cell Lines and Cultures

The HCC cell lines Huh7, LM3 and SMMC-7721 were purchased from the Chinese Academy of Sciences Committee Type Culture Collection Cell Bank. The three cell lines were cultured in high glucose Dulbecco’s modified Eagle’s medium (DMEM-h; Thermo, China) supplemented with 10% fetal bovine serum, 100 U/ml penicillin, and 100 µg/ml streptomycin in a humidified incubator at 37°C in 5% CO_2_.

### 2.2 Drugs and Antibodies

5-FU and Sal were purchased from Sigma Aldrich (St. Louis, MO, USA). A stock solution of 25 mg/ml 5-FU and 25 mM Sal which prepared using dimethyl sulfoxide (DMSO) were stored in the dark at −20°C. The final 5-FU and Sal concentrations used in the experiments were prepared from the stock solutions by dilution in DMEM-h. The antibodies CD133 (Miltenyi, Germany) and EPCAM (eBioscience, USA) were used for flow cytometric analysis, β-actin(Santa Cruz, CA, USA), E-cadherin (Abcam, USA), vimentin (Abcam, USA), p-GSK-3β-Tyr216(Santa Cruz, CA, USA), p-β-catenin (Cell Signaling Technology, USA) and active β-catenin (Cell Signaling Technology, USA) for Western blotting, active β-catenin (Cell Signaling Technology, USA) for immunofluorescence, CD133 (Bioss, China), EPCAM (eBioscience, USA), E-cadherin (Abcam, USA), vimentin (Abcam, USA) and active β-catenin (Cell Signaling Technology, USA) for immunohistochemistry.

### 2.3 Cell Viability

The HCC cell lines Huh7, LM3 and SMMC-7721were plated in 96-well plates (100 µl media per well). One day after seeding, 5-FU (0 ug/ml, 2 ug/ml, 4 ug/ml, 8 ug/ml, 16 ug/ml) and Sal (0 µM, 2 µM, 4 µM, 8 µM, 16 µM) were added in five replicates to each cell population. Cell viability was measured after 24 h, 48 h and 72 h using the MTT assay and a microplate reader at 490 nm. A calibration curve was prepared using the data obtained from wells that contained a known number of viable cells.

### 2.4 Combination Analysis

To evaluate the pharmacological interactions of the different combinations of drugs, we used the method of Chou et al [19]. Briefly, synergism, additivity or antagonism in the different combinations was calculated on the basis of the multiple drug effect equation and quantitated by the combination index (CI), where CI = 1 indicates that the two drugs have additive effects, CI<1 indicates more than additive effects (“synergism”) and CI>1 indicates less than additive effects (“antagonism”). The CI was calculated based on: CI = (D)1/(Dx)1+(D)2/(Dx)2+(D)1(D)2/(Dx)1(Dx)2, where (Dx)1 and (Dx)2 are the doses of drug 1 and drug 2, alone, inhibiting ‘x%’, whereas (D1) is the dose of drug 1 in combination, and (D2) the dose of drug 2 in combination that gives the experimentally observed ‘x’ inhibition. Because our aim was to achieve maximal effect of the drugs tested on cancer cells, a mean CI was calculated from data points with fraction affected (Fa) >0.5. Fa<0.5 indicated lower growth inhibition and a large fraction of the cell population indicated growth. Fa<0.5 was therefore considered irrelevant. Furthermore, we evaluated the drug dose in a synergistic combination. This was designated as the dose reduction index (DRI): (DRI)1 = (Dx)1/(D)1 and (DRI)2 = (Dx)2/(D)2 where DRI>1, which showed that combinations could result in reduced drug doses compared with the doses for each drug alone. Classical isobolograms were also constructed by plotting drugs concentrations (alone and in combination) that inhibits 50%, 60%, 70% HCC cell viability. First, the concentrations of 5-FU and Sal required to produce a defined single-agent effect, when used as single agents, are placed on the *x* and *y* axes in a two-coordinate plot, corresponding to (C_5-FU_, 0) and (0, C_Sal_), respectively. The line connecting these two points is the line of additivity. Second, the concentrations of the two drugs used in combination to provide the same effect, denoted as (C_5-FU_, C_Sal_), are placed in the same plot. Synergy, additivity, or antagonism are indicated when (C_5-FU_, C_Sal_) is located below, on, or above the line, respectively.

### 2.5 Flow Cytometric Analysis

Analysis of apoptosis: Huh7, LM3 and SMMC-7721 cells were plated in 6-well plates. After 48 h, control cells, 5-FU-treated cells (8 ug/ml,) Sal-treated cells (4 uM) and 5-FU plus Sal treated cells were collected, washed twice in cold PBS, mixed in 100 ml of binding buffer, and incubated at room temperature for 15 min with an annexin-V/PI (BD Biosciences) double staining solution. Stained cells were analyzed by flow cytometry and the percentage of apoptotic cells was calculated using ModFitLT software (Verity Software House).

Analysis of CD133(+) EPCAM(+) cells: Huh7 cells were plated (100,000 cells per well) in six-well plates. After 48 h, cells from the control group, the 5-FU group, the Sal group and the 5-FU plus Sal group were collected, and washed twice in cold phosphate buffered saline (PBS). Dissociated cells were stained with PE (phycoerythrin)-conjugated CD133 antibody and FITC (fluorescein isothiocyanate)-conjugated EPCAM antibody, and then co-incubated for 30 min at 4°C. Mouse IgG1-phycoerythrin was used as an isotype control antibody. Dead cells were eliminated with 7-aminoactinomycin D. The labeled cells were analyzed by the BD FACSVantage system (BD Biosciences, San Jose, CA, USA) in accordance with the manufacturer’s protocols. Gating was implemented on the basis of negative control staining profiles.

### 2.6 Colony/Sphere Formation Assays

Huh7 cells treated with DMSO vehicle, 5-FU, Sal and Sal plus 5-FU were resuspended as single cells in 1.2% agar (Sigma-Aldrich, St. Louis, MO, USA) and diluted with ddH2O. This was overlaid on a base of 0.6% agar diluted with ddH2O. Both the top and base layers were mixed with DMEM-h and 20% FBS. After 10 days, the number of colonies which developed within each well were counted and photographed under a microscope using an inverted digital camera.

### 2.7 Animal Experiments

Animal experiments were performed on 6-week-old male nude mice (athymic, BALB/C nu/nu). A high standard of ethics was applied in carrying out the investigations. The mice were housed in a standard animal laboratory with free access to water and food. They were kept under constant environmental conditions with a 12-hour light-dark cycle. All operations were performed under aseptic conditions. All procedures were approved by the Animal Care and Use Committee of Shanghai Tongji University. The animal experiment permit number is SYXK (Shanghai) 2011-0111.

### 2.8 Treatments in Mouse Xenograft Models

Huh7 (5×106 cells) in 100 µl DMEM-h and 100 µl Matrigel (Becton Dickinson, Bedford, MA, USA) were injected subcutaneously into each mouse. When the tumor volume was approximately 100 mm^3^, the animals were randomly divided into four groups (saline, 5-FU, Sal and Sal plus 5-FU), and intraperitoneally injected with test reagents or saline daily for 4 weeks.

### 2.9 Anticancer Drug In vivo Analysis

Data were evaluated using the National Cancer Institute guidelines for assessment of anticancer drug effects in subcutaneously growing human tumor xenografts [20]–[22]. The anti-tumor effect was observed by measuring tumor diameter in the test animals twice per week, and tumor volume (TV) was calculated as: TV = 1/2×a×b^2^ (a, b denote the long and short diameters, respectively). Relative tumor volume (RTV) was calculated based on the measured results: RTV = Vt/V_0_ (V_0_: the tumor volume at initial administration, Vt: the tumor volume at each time measurement). Anti-tumor activity was evaluated by the relative tumor growth rate T/C (%) = TRTV/CRTV×100%, (TRTV: treatment group RTV; CRTV: negative control group RTV). Then tumor weight was used to evaluate the efficacy of the drugs. Following administration, the animals were killed, and the tumor block was dissected and weighed. The tumor weight evaluation formula: tumor growth inhibition rate  =  (the average tumor weight in the administration group - the average tumor weight in the negative control group)/the average tumor weight in the negative control group×100%.

### 2.10 Immunofluorescence

Huh7 cells treated with DMSO vehicle, 5-FU, Sal or Sal plus 5-FU were planted on poly-L-lysine coated glass coverslips, fixed with cold acetone for 1 h, and permeabilized in 0.5% Triton X-100 (Sigma-Aldrich) for 10 min. The cells were blocked with bovine serum albumin (BSA) in PBS and incubated with primary antibodies (anti-active β-catenin) overnight at 4°C. The following morning, the slides were washed with PBS and incubated with appropriate fluorescein isothiocyanate-conjugated secondary antibody for 1 h. The cells were washed and incubated with DAPI (Invitrogen, Carlsbad, CA, USA) for nuclear staining, washed and mounted with propyl gallate under glass coverslips. The slides were visualized with a scanning laser microscope (Zeiss 710, Germany).

### 2.11 Immunohistochemistry

Tumor tissues from the control group, 5-FU-treated group, Sal-treated group, and 5-FU plus Sal-treated group were analyzed by immunohistochemistry. Sections (4 µm thick) from paraffin-embedded tumors were deparaffinized and rehydrated using xylene and ethanol, respectively, and immersed in 3% hydrogen peroxide solution for 10 min to block endogenous peroxidases. Sections were boiled for 30 min in 10 mM citrate buffer solution (pH 6.0) for antigen exposure. Slides were incubated for 45 min with 5% BSA and incubated overnight at 4°C with the primary antibodies against CD133, EPCAM, E-cadherin, vimentin and active β-catenin. These specimens were incubated for 45 min at 37°C with the appropriate peroxidase-conjugated secondary antibody and visualized using the Real Envision Detection Kit (Gene Tech Shanghai Company Limited, China) following the manufacturer’s instructions.

### 2.12 Reverse Transcription-Polymerase Chain Reaction (RT-PCR) and Real-time PCR

Total RNA was extracted and first-strand cDNA was synthesized using the Omniscript RT kit (QIAGEN, Gaithersburg, MD, USA), with 2000 ng RNA (per 20 µl reaction) and oligo (dT) primers. cDNA was used in real-time PCR reactions to analyze E-cadherin, vimentin, and β-actin expression. Primers used in the PCR reactions are listed in **Table 1**. PCR reactions were amplified for 40 cycles. Each cycle consisted of denaturation for 1 min at 94°C, annealing for 1 min at 60°C, and polymerization for 2 min at 72°C. PCR products were quantified using the Molecular Analyst software (Bio-Rad, Hercules, CA, USA). The ratio of each gene *vs* β-actin was calculated by standardizing the ratio of each control to the unit value.

**Table 1:** Real-time PCR Primer Sequences.

Gene		Primer sequence (5′→3′)
E-cadherin	Forward	ATTTTTCCCTCGACACCCGAT
	Reverse	TCCCAGGCGTAGACCAAGA
Vimentin	Forward	AGTCCACTGAGTACCGGAGAC
	Reverse	CATTTCACGCATCTGGCGTTC
β-actin	Forward	CTGGAACGGTGAAGGTGACA
	Reverse	AAGGGACTTCCTGTAACAATGCA

### 2.13 Western Blot Assays

Sample proteins were electrophoretically separated on a 10% polyacrylamide gel at 80 volts. The proteins were transferred to a polyvinylidene difluoride membrane. Membranes were blocked for 60 min with a 5% (v/v) milk solution prepared in PBS. The membranes were incubated overnight at 4°C with 1∶500 dilutions of the primary antibodies (against E-cadherin, vimentin, p-GSK-3β (Tyr216), p-β-catenin, active-β-catenin and β-actin). Membranes were washed three times for 5 min each with Tween 20 (1∶1000) in PBS and incubated for 45 min with the appropriate peroxidase-conjugated secondary antibody (1∶1000 in PBS). Membranes were washed three times with Tween 20-PBS, for 10 min each, and were developed using the Odyssey Two-color Infrared Laser Imaging System (Li-Cor, Lincoln, NE, USA). The signal generated by β-actin was used as an internal control.

### 2.14 Statistical Analyses

SPSS 17.0 software (IBM, Armonk, NY, USA) was used for statistical analyses. Experiments were repeated at least three times. Unless otherwise stated, data are expressed as the mean ± standard deviation. The real-time PCR data were 2-△△Ct transformed before analysis and were analyzed using analysis of variance (ANOVA). The results of MTT assay, flow cytometric analysis, colony/sphere formation assay, tumor volume and weight analysis, and western blots were analyzed using ANOVA. If the result of the ANOVA was significant (*p*<0.05 versus control), pairwise comparisons between the groups were performed using a post hoc test (S-N-K procedure). In all cases, *p*<0.05 was considered statistically significant.

## Results

### 3.1 Single Drug Treatment with 5-FU or Sal

**Figure 1:**
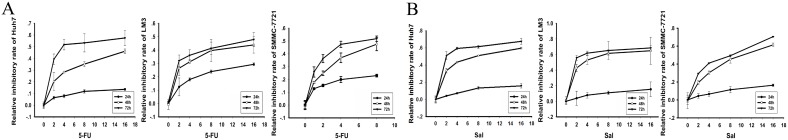
Growth inhibition curves for HCC cell lines Huh7, LM3, and SMMC-7721. 5-FU (A) and Sal (B) inhibit HCC cell proliferation. Huh7, LM3, and SMMC-7721 (5×10^4^ cells/ml) were treated with Sal and 5-FU for various times (24, 36, and 48 h). Cell viability was determined using the MTT assay. The data show that Sal and 5-FU exposure reduced Huh7, LM3, and SMMC-7721 cell viability in a dose- and time-dependent manner.

To test the effects of 5-FU on growth of HCC cells, HCC cell lines Huh7, LM3 and SMMC-7721 were treated with increasing concentrations of 5-FU (0, 2, 4, 8 and 16 ug/ml) for 24 h, 48 h and 72 h. As shown in **Fig. 1A**, cell growth was inhibited by 5-FU in a dose- and time-dependent manner. We subsequently evaluated the effect of Sal (0, 2, 4, 8 and 16 µM) for 24 h, 48 h and 72 h on cell growth, and found that Sal was effective in inhibiting cell growth of all cell lines tested (**Fig. 1B**). These results indicate that 5-FU or Sal was an effective inhibitor of HCC cell growth as a single agent. Subsequent studies were undertaken to examine if the cells treated with Sal combined with 5-FU were more sensitive to the cytotoxic effect of 5-FU.

### 3.2 Combination Treatment with Sal and 5-FU

**Figure 2:**
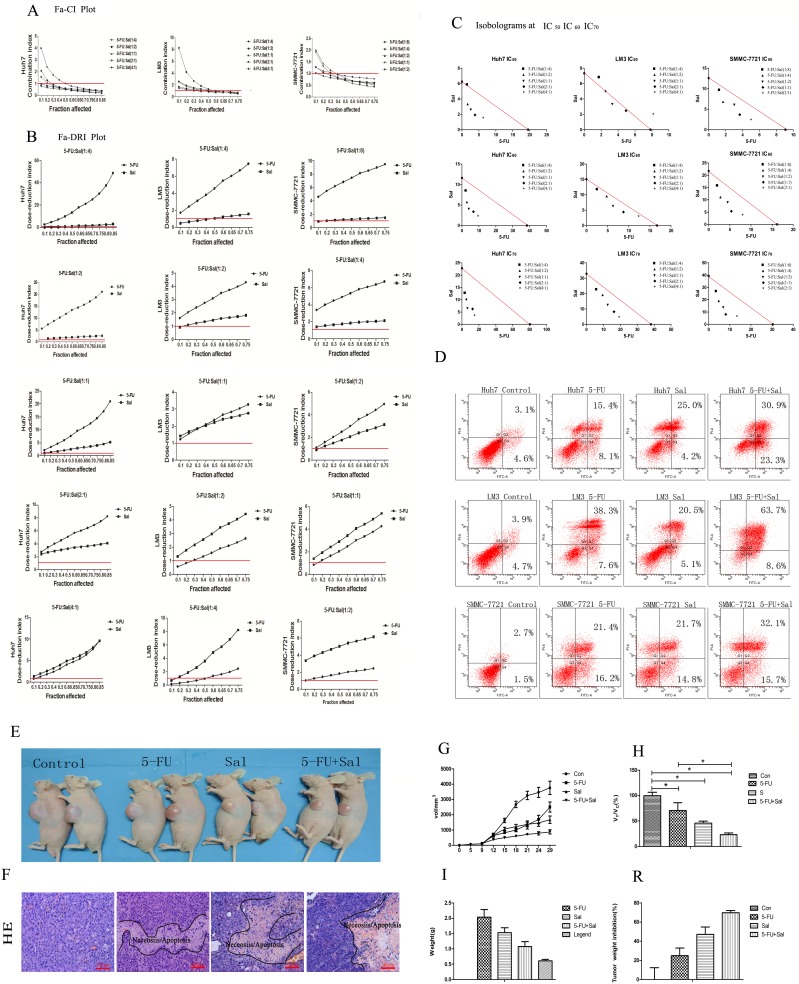
Combination treatment with 5-FU and Sal. (A–D) Illustrative Fa-CI and Fa-DRI plots for the combination of 5-FU and Sal using different fixed drug ratios. (A) CI values were calculated from each Fa for HCC cell lines Huh7, LM3, and SMMC-7721. Average synergism (CI<1) at Fa>0.5 for all three HCC lines. (B) DRI values were calculated from each Fa for HCC cell lines Huh7, LM3, and SMMC-7721. The 5-FU and Sal chemotherapeutic doses may be significantly reduced (DRI>1) for combinations that are synergistic at Fa>0.5 for all three HCC lines. (C) Isobologram analysis at IC_50_, IC_60_ and IC_70_ for the combinations of HCC cell lines Huh7, LM3, and SMMC-7721. The results indicats synergy, additivity or antagonism when the points are located below, on or above the line, respectively. We can see that (C_5-FU_, C_Sal_) is located below the line (synergy) at IC _60_, IC _70_ for HCC cell lines Huh7, LM3 and SMMC-7721. (D) The combination effect of Sal and 5-FU on apoptosis effects were evaluated by flow cytometric analysis. The results showed that combination therapy increased apoptosis of HCC cell lines Huh7, LM3 and SMMC-7721 significantly. (E–R) Combination treatments in the *in vivo* models (E) Subcutaneous tumor volume following combination therapy was reduced compared to that of the other three groups (two representative mice in each group). (F) HE staining showed the area of apoptosis and necrosis induced by drugs in tumor tissue of treatment group. (G) The tumor growth curve showed that tumor growth rate following combination therapy was slower than that of the other three groups. (H) The relative tumor proliferation rate, V_Treatment_/V_Control_, showed that proliferation rate of the combination therapy group was slower than that of the other three groups. (* ***p*** **<0.05**)s. (I) In the combination therapy group, tumor blocks weighed lighter than those of the other three groups (* ***p*** **<0.05**). (R) The tumor growth inhibition rate indicated that the combination therapy significantly inhibited tumor growth than the other three groups (* ***p*** **<0.05**).

The effect of Sal combined with 5-FU on cell viability was investigated using the MTT assay. For these studies, HCC cell lines Huh7, LM3 and SMMC-7721 were treated with 5-FU (0, 2, 4, 8, and 16 ug/ml), Sal (0, 2, 4, 8, and 16 µM), or Sal plus 5-FU for 48 h. Viable cells were evaluated using the MTT assay. Treatment of HCC cells with 5-FU plus Sal for 48 h resulted in a decrease in cell viability which was greater than either 5-FU or Sal alone (**Table 2**). Fraction affected (Fa) values (indicating the fraction of cells inhibited after drug exposure) were obtained after exposure of the cells to a series of drug concentrations. To indicate the effects at different Fa values, the CI (combination index) and DRI (dose reduction index) values were calculated for each Fa. **Fig. 2A** shows the Fa-CI plots illustrating the effects of Sal and 5-FU at different fixed drug ratios, and demonstrates synergism (CI<1) at Fa>0.5 for HCC cell lines Huh7, LM3 and SMMC-7721. As expected, synergism corresponding to CI<1 always yielded a favorable DRI (>1) for both drugs. The Fa-DRI plots are shown in **Fig. 2B**, and indicate that chemotherapeutic doses of 5-FU may be significantly reduced for combinations with Sal that are synergistic at Fa>0.5. Classical isobolograms were shown in Fig. 2C, we can see that (C_5-FU_, C_Sal_) is located below the line (synergy) at IC_60_, IC_70_ for HCC cell lines Huh7, LM3 and SMMC-7721. At last the combination effect of Sal and 5-FU on apoptosis effects were evaluated by flow cytometric analysis. The results (Fig. 2D) showed that combination therapy increased apoptosis of HCC cell lines Huh7, LM3 and SMMC-7721 significantly.

**Table 2:** Relative inhibitory by 5-FU, Sal and their combination for HCC cell lines.

Huh7	5-FU(0 µg/ml)	5-FU(2 µg/ml)	5-FU(4 µg/ml)	5-FU(8 µg/ml)	5-FU(16 µg/ml)
Sal(0 µM)	0±0.027	0.203±0.077	0.282±0.050	0.351±0.022	0.461±0.019
Sal(2 µM)	0.335±0.027	0.485±0.069	0.510±0.029	0.559±0.051	0.591±0.082
Sal(4 µM)	0.433±0.079	0.549±0.014	0.568±0.078	0.622±0.063	0.714±0.037
Sal(8 µM)	0.509±0.061	0.582±0.163	0.629±0.094	0.674±0.044	0.738±0.029
Sal(16 µM)	0.597±0.009	0.649±0.102	0.747±0.101	0.775±0.029	0.873±0.072
**LM3**	**5-FU(0 µg/ml)**	**5-FU(2 µg/ml)**	**5-FU(4 µg/ml)**	**5-FU(8 µg/ml)**	**5-FU(16 µg/ml)**
Sal(0 µM)	0±0.005	0.292±0.056	0.462±0.015	0.518±0.082	0.568±0.060
Sal(2 µM)	0.320±0.048	0.423±0.030	0.451±0.154	0.495±0.043	0.596±0.009
Sal(4 µM)	0.420±0.054	0.508±0.088	0.531±0.018	0.607±0.018	0.660±0.015
Sal(8 µM)	0.520±0.022	0.527±0.013	0.582±0.072	0.628±0.054	0.688±0.015
Sal(16 µM)	0.603±0.173	0.636±0.047	0.645±0.078	0.680±0.072	0.736±0.025
**SMMC-7721**	**5-FU(0 µg/ml**	**5-FU(1 µg/ml)**	**5-FU(2 µg/ml)**	**5-FU(4 µg/mlg)**	**5-FU(8 µg/ml)**
Sal(0 µM)	−0.001±0.008	0.178±0.006	0.248±0.018	0.369±0.038	0.475±0.050
Sal(2 µM)	0.193±0.021	0.223±0.087	0.336±0.105	0.452±0.116	0.552±0.124
Sal(4 µM)	0.303±0.019	0.397±0.049	0.432±0.061	0.539±0.029	0.600±0.014
Sal(8 µM)	0.453±0.042	0.460±0.086	0.524±0.050	0.572±0.011	0.690±0.028
Sal(16 µM)	0.478±0.021	0.553±0.067	0.601±0.038	0.673±0.060	0.721±0.036

Cells were exposed to a concentration range of 5-FU, Sal and their combination for 48 h.

Values (relative inhibitory) are means ± standard error of the mean (SE) for 3–5 experiments.

To explore the effects of the combination of 5-FU and Sal *in vivo*, we established mouse xenograft models using Huh7 cells. Saline, 5-FU (8 mg/kg) [23], Sal (4 mg/kg) [17] and 5-FU (8 mg/kg) plus Sal (4 mg/kg) were used for the *in vivo* experiments. There were six mice of each group. Dynamic observations of the anti-tumor effects of the test substances were carried out for 4 weeks. It can be seen in **Fig. 2E** that the subcutaneous tumor volume (we choose two representative mice in each group) was reduced in the combination therapy group compared to the other three groups. HE staining (**Fig. 2F**) showed the area of apoptosis and necrosis induced by drugs in tumor tissue of treatment group. Details of the evaluation criteria and methods are shown in the Materials and Methods section. The anti-tumor effect was observed by measuring tumor diameter in the test animals twice per week, and the tumor growth curve is shown in **Fig. 2G**. The result showed that tumor growth rate in the combination therapy group was slower than that in the other three groups. The relative tumor proliferation rate V_Treatment_/V_Control_ (**Fig. 2H**) in the combination therapy group was slower (*p **<0.05**) than that in the other three groups. Tumor weight was calculated to evaluate drug efficacy and is shown in **Fig. 2I**. The tumor blocks in the combination therapy group weighed lighter (*p **<0.05**) than those in the other three groups. Tumor growth inhibition rate (**Fig. 2R**) was greater in the combination therapy (*p **<0.05**). These findings suggested that 5-FU combined with Sal was effective and tolerable as a novel therapeutic modality for HCC.

### 3.3 Effects of 5-FU, Sal and their Combination on the Cancer Stem Cell Properties of HCC Cells

**Figure 3:**
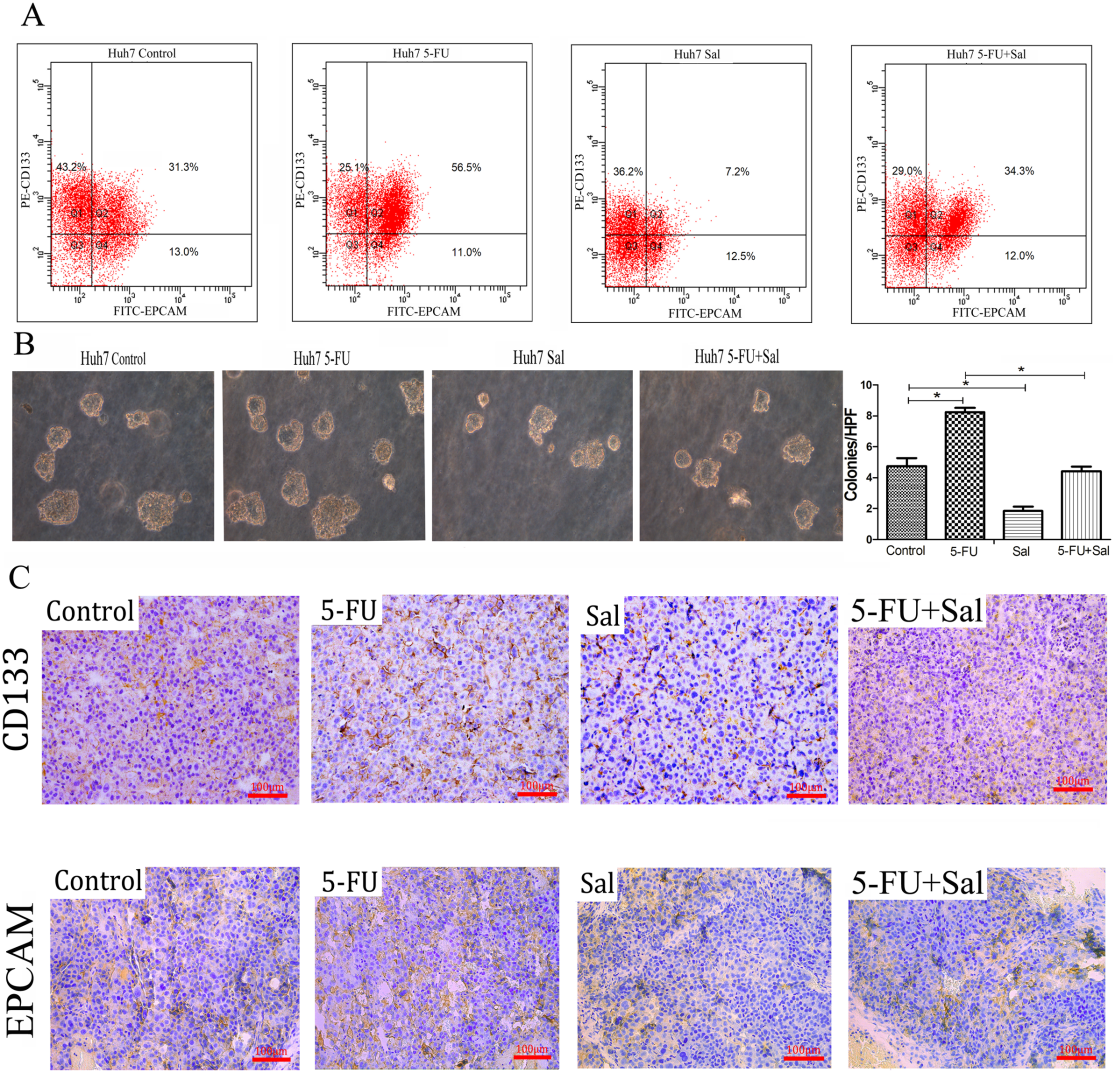
Effects of 5-FU, Sal and their combination on cancer stem cell properties in HCC cells. (A) Flow cytometry assays showed that treatment with 5-FU increased the proportion of the CD133+ EPCAM+ Huh7 cell subpopulation compared with the control group. In contrast, treatment with Sal reduced this proportion compared with the control group. 5-FU combined with Sal reduced this proportion compared with the 5-FU group (*p **<0.05**). (B) Colony-forming assays were performed to measure the proliferative ability of single cancer cells. The number of colonies increased in the 5-FU treatment group compared with the control group, decreased in the Sal treatment group compared with the control group. The number of colonies was significantly lower in the Sal plus 5-FU combination group compared with the 5-FU treatment group (* ***p*** **<0.05**). (C) Immunohistochemistry indicates CD133+ and EPCAM+ expression in the tumors of mouse xenograft models. (Magnification is 200×).

EpCAM and CD133 have been used as cancer stem cells (CSCs) markers in HCC. Research has shown that both EpCAM and CD133 surface markers were more representative for CSC s in HCC Huh7 cells [24]. We performed flow cytometry to determine the effects of 5-FU and Sal on the proportion of HCC cells with the CD133(+) EPCAM(+) antigenic phenotype (**Fig. 3A**). Treatment with 5-FU increased the proportion of the CD133(+) EPCAM(+) cell subpopulation from 27.77±4.72% (vehicle-treated controls) to 53.5±3.17% (* ***p*** **<0.05**). In contrast, treatment with Sal reduced this proportion from 27.77±4.72% (vehicle-treated controls) to 6±1.70% (* ***p*** **<0.05**). There was a significant decrease in the CD133(+) EPCAM(+) cell subpopulation in the 5-FU plus Sal combination therapy group compared with 5-FU monotherapy (26.73±8.27% *vs* 53.57±3.17, * ***p*** **<0.05**). We know that cancer stem cells have a strong proliferative ability, thus, colony-forming assays (**Fig. 3B**) were performed to measure the proliferative ability of single cancer cells. Huh7 cells were treated with DMSO vehicle, 5-FU (44 ug/ml), Sal (2 µM) and Sal plus 5-FU for 96 h. In all cases, colonies were visible after 10 days. The number of colonies increased in the 5-FU treatment group (8.25±0.25 colonies/high power field (HPF)), and decreased in the Sal treatment group (1.83±0.29 colonies/HPF), relative to vehicle-treated controls (4.75±0.05 colonies/HPF) (*p **<0.05**). The number of colonies was significantly lower in the Sal plus 5-FU combination group (4.42±0.29 colonies/HPF) compared with the 5-FU treatment group (8.25±0.25 colonies/HPF) (*p **<0.05**).

Finally, the expression of CD133 and EPCAM (**Fig. 3C**) were evaluated in the tumors of mouse xenograft models by immunohistochemistry (200×). The expression of CD133 was increased in the 5-FU group compared with the saline group. In contrast, Sal treatment reduced the expression of CD133 compared with the saline group, and 5-FU combined with Sal reduced the proportion of CD133 compared with the 5-FU treatment group. Similar results were obtained for the expression of EPCAM.

### 3.4 Sal Altered Epithelial-Mesenchymal Transition (EMT) Induced by 5-FU

**Figure 4:**
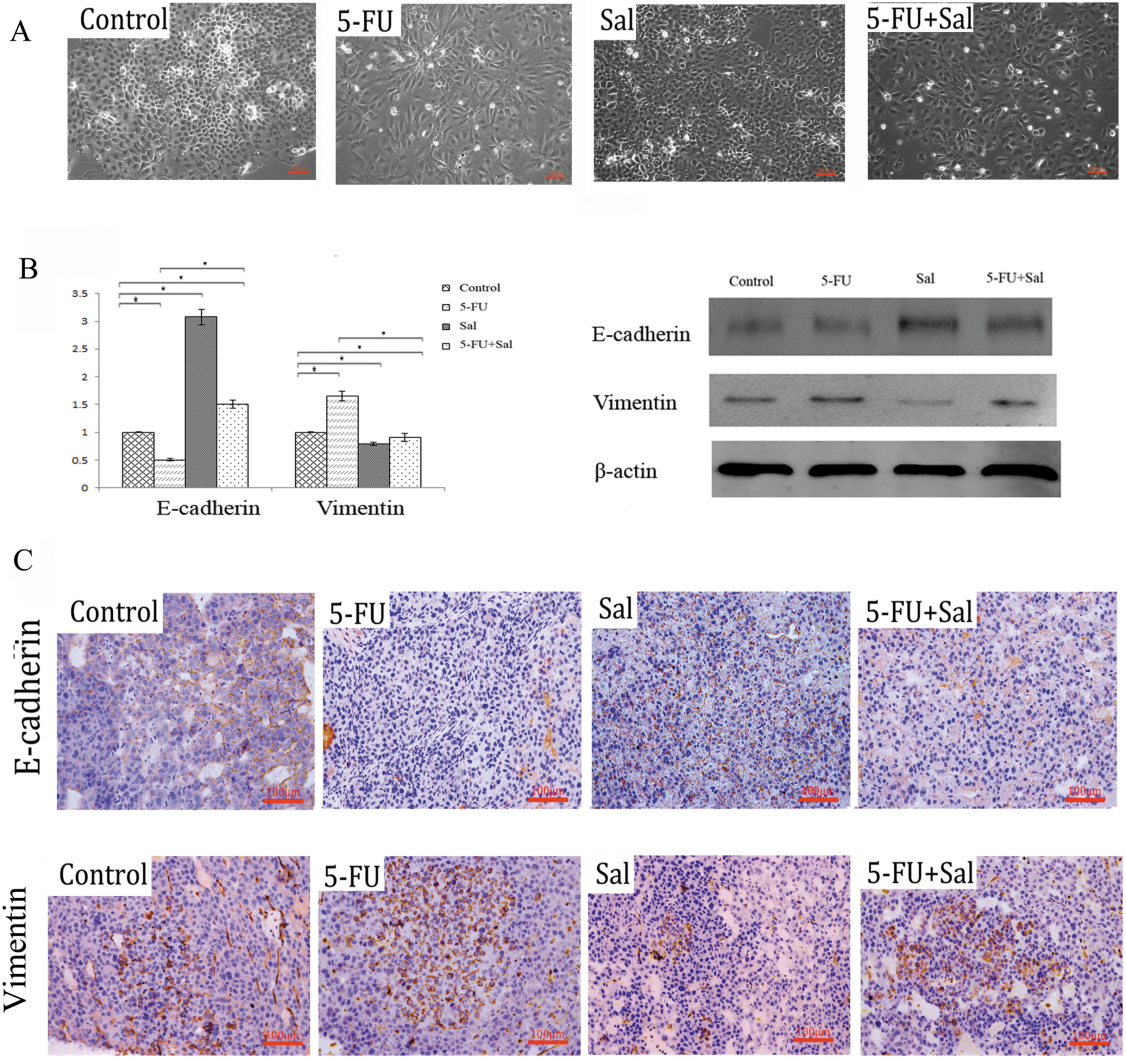
Effect of 5-FU, Sal, and 5-FU combined with Sal on the epithelial-mesenchymal transition (EMT)-related process. (A) Morphological changes after the indicated treatment in Huh7 cells (Magnification 200×). (B) Real-time PCR was performed to examine mRNA expression of EMT-related genes (E-cadherin, vimentin) (* ***p*** **<0.05**). Western blot was performed to examine protein expression of EMT-related genes (E-cadherin, vimentin). (C) Immunohistochemistry indicates E-cadherin and vimentin expression in the tumors of mouse xenograft models (Magnification 200×).

Many laboratories have shown that EMT can endow cells with stem cell-like characteristics [25], and similar results have been found in hepatoma cells [26]. Emerging evidence has associated chemo-resistance with acquisition of EMT which is involved in acquired resistance to 5-FU [27]. To determine whether Sal altered the EMT process induced by 5-FU, we evaluated EMT in Huh7 cells treated with vehicle control, 5-FU, Sal and Sal plus 5-FU. Following treatment with 5-FU, Huh7 cellular morphology was converted to a diffuse fibroblast-like morphology, characteristic of EMT, as compared with untreated cells. Cells treated with Sal were round and cells in the combination treatment group were rounder than those in the 5-FU group (**Fig. 4A**). In order to further investigate EMT in Huh7 cells, we examined the more common markers of EMT, using Real time-PCR and western blot. The data showed that 5-FU induced EMT in Huh7 cells by down-regulation of E-cadherin and up-regulation of vimentin expression. In contrast to 5-FU, Sal inhibited EMT by upregulating the expression of E-cadherin and down-regulating the expression of vimentin. In addition, 5-FU combined with Sal demonstrated that Sal altered EMT induced by 5-FU (**Fig. 4B** **,** *p<0.05). Similar results were observed in tumors in mouse xenograft models by immunehistochemistry (200×) (**Fig. 4C**).

### 3.5 Sal Blocks the Wnt-β-catenin Pathway

**Figure 5:**
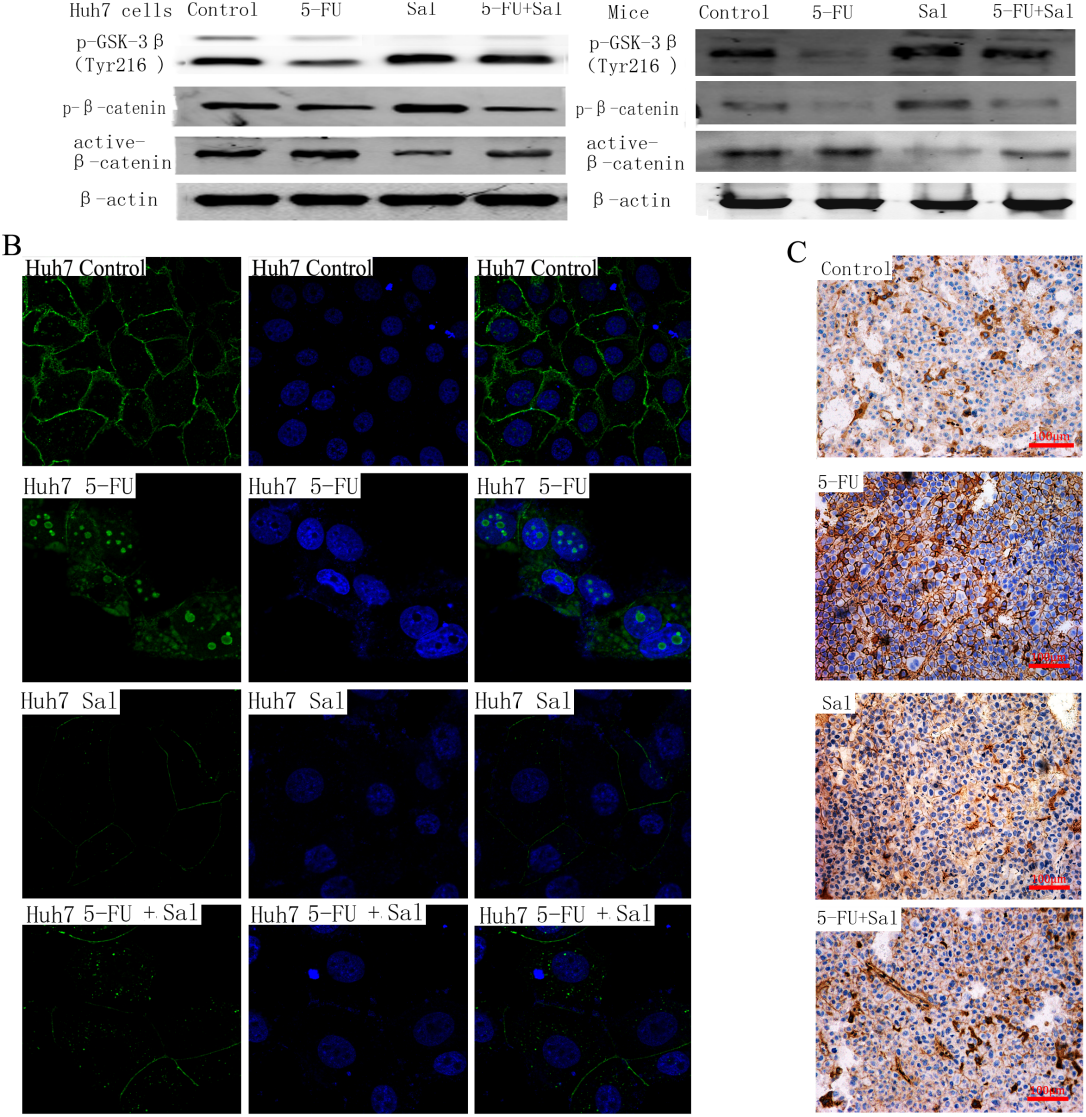
Translocation of β-catenin. (A) The protein expression of p-GSK-3β (Tyr 216) which is active-GSK-3β, p-β-catenin which is inactive β-catenin and active-β-catenin were detected by western-blot in vitro and in vivo. Compared to 5-FU group, p-GSK-3β (Tyr216) expression of Sal group and combination therapy group were significantly up-regulated and we found the similar changes in p-β-catenin protein. Decreased expression of active β-catenin protein were observed in Sal group and combination therapy group compared to 5-FU alone group. (B) Changes in cellular localization of active β-catenin in Huh7 cells. Active β-catenin cellular localization was evaluated by indirect immunofluorescence. Immunofluorescence were labeled of active β-catenin in Huh7 cells (untreated, treated with 5-FU, Sal and Sal plus 5-FU) for 48 h. Nuclei were stained with DAPI, and regions were merged to assess signal colocalization. Magnification is 630×. In the control condition, active β-catenin is present in the cytomembrane and cytoplasm. In the 5-FU treated groups, active β-catenin preferentially accumulates in the nuclear and perinuclear region. In contrast, cells treated with Sal showed preferential localization of active β-catenin in cytomembrane, altering the translocation of active β-catenin to the nucleus. Cells treated with the combination of 5-FU and Sal showed decreased accumulation of β-catenin in the nuclear and perinuclear region compared with the 5-FU treated groups. (C) Immunohistochemistry indicates similar results for β-catenin in the tumors of mouse xenograft models. Magnification is 200×.

Wnt/beta-catenin signaling contributes to the activation of tumorigenic liver progenitor cells [28], [29]. and EMT [30], [31]. β-catenin is a key component of the Wnt/β-catenin signaling pathway. In canonical Wnt signaling pathways, Gsk-3β is the upstream adjustment factor of β-catenin and can compose a complex with APC and axin to phosphorylation β-catenin, leading toβ-catenin degradation. Here we examined the protein expression of p-GSK-3β(Tyr216) which is the active GSK-3β, p-β-catenin which is the inactive β-catenin and active β-catenin *in vitro* and *in vivo* by western-blot (Fig. 5A). Compared to 5-FU group, p-GSK-3β (Tyr216) expression of Sal group and combination therapy group were significantly up-regulated and we found the similar changes in p-β-catenin protein. Decreased expression of active β-catenin protein were also observed in Sal group and combination therapy group in comparison with 5-FU alone group. In addition to examining the change of protein expression of avtive β-catenin, the changes in active β-catenin localization were also obversed following treatment with 5-FU, Sal, and Sal plus 5-FU by indirect immunofluorescence detection in Huh7 cells (Fig. 5B). In the control condition, active β-catenin was present in the cytomembrane and cytoplasm. However, in the 5-FU treated group, active β-catenin preferentially accumulated in the nuclear and perinuclear region which promoted the activation of Wnt/β-catenin signaling pathway. In contrast, in the cells treated with Sal, active β-catenin was preferentially accumulated in the cytomembrane and down-regulated expression which meant translocation of active β-catenin to the nucleus was blocked. Interestingly, treatment with 5-FU and Sal showed decreased accumulation of active β-catenin in the nuclear area compared with the 5-FU group. We also observed comparable results following immunohistochemical analysis of tumors in mouse xenograft models (Fig. 5C).

## Discussion

Once diagnosed with HCC, only 30–40% of patients are deemed eligible for curative treatment, including surgical resection, liver transplantation, and chemoembolization. Most patients will receive some form of chemotherapy in the hope of prolonging life. Emerging data has indicated that HCC CSCs are resistant to conventional chemotherapy such as 5-FU. Targeting CSCs therapeutically is likely to be challenging, because both bulk tumor cells and CSCs must be eliminated, potentially demanding combination drug therapies. Sal, an antibiotic used to kill bacteria, fungi and parasites, has recently been shown to selectively deplete human breast cancer stem cells [14] and colorectal cancer stem cells [22]. In previous experiments we found that Sal down-regulated the CD133+ cell subpopulation in HCC cells [17]. Taking into account the characteristics of Sal, in this study we used three HCC cell lines and a nude mouse subcutaneous tumor model to determine if the combination of 5-FU and Sal could enhance the sensitivity of HCC cells to conventional chemotherapy such as 5-FU. We found that combination therapy with Sal and 5-FU had a synergistic antitumor effect against liver tumors both *in vitro* and *in vivo*.

We next explored whether Sal affected drug resistance induced by 5-FU. The cell surface markers, CD133 and EPCAM, are frequently used to identify CSCs in various tumors, including HCC [32], [33]. In addition, research [24] has shown that the CD133(+) EpCAM(+) phenotype is precisely represented by CSCs in Huh7 cells. In our study, the results showed that Sal combined with 5-FU decreased the proportion of CD133(+) EpCAM(+) which were increased in the 5-FU alone group of Huh7 cells. Sal combined with 5-FU also inhibited the expression of CD133 and EPCAM respectively in subcutaneous tumor tissue of nude mice. Another observed effect of treatment on HCC CSCs was decreased clonogenicity. These effects may be due to Sal combined with 5-FU reducing the proportion of CD133(+) EpCAM(+) cell subpopulations within Huh7 cells, suggesting that inhibition of tumorigenic/proliferative ability of HCC CSCs by Sal was associated with sensitization of HCC cells to 5-FU.

Many laboratories have shown that EMT is related to chemotherapy drug resistance as it can endow cells with stem cell-like characteristics [34]–[36], and similar results were obtained with hepatoma cells [37], [38]. In the present study, we found that 5-FU induced Huh7 cells to mesenchymal-like cancers *in vitro* and *in vivo*, by reducing E-cadherin and increasing vimentin expression, however, treatment with Sal plus 5-FU could reverse EMT induced by 5-FU.

Studies have shown that dysregulation of the Wnt/β-catenin signaling pathway is involved in cancer chemoresistance [39], contributes to the induction of EMT [40] and promotes stem cell maintenance [41]. The classical Wnt signaling pathway is mediated by β-catenin, and accumulated nuclear localization of active β-catenin increases resistance to 5-FU in HCC cells. In the present study, our results demonstrated that Sal alone and Sal combined with 5-FU down-reglated the expression of active β-catenin by down-reglating p-GSK-3β (Tyr216) which is active GSK-3β and induced preferential periplasmic membrane localization of active β-catenin in comparison with 5-FU alone, The result indicated combination therapy revised the activation of Wnt/β-catenin signaling pathway induced by 5-FU.

In conclusion, our current findings show that Sal potentiates the antitumor effects of 5-FU by down-regulating CSCs in HCC cells. Strategies to modulate EMT by blocking the translocation of active β-catenin to the nucleus might play a role in the down-regulation of CSCs. In addition to 5-FU, there are many drug resistance processes caused by CSCs enrichment such as those involved in resistance to cisplatin [42] and cyclophosphamide [43]. This study provides us with a new approach to reverse drug resistant for the treatment of patients with HCC.
